# Simplicity From Complexity in Vertebrate Behavior: Macphail (1987) Revisited

**DOI:** 10.3389/fpsyg.2020.581899

**Published:** 2020-10-28

**Authors:** Stephen B. Fountain, Katherine H. Dyer, Claire C. Jackman

**Affiliations:** ^1^Department of Psychological Sciences and Brain Health Research Institute, Kent State University, Kent, OH, United States; ^2^Department of Psychological Sciences, Kent State University, Kent, OH, United States

**Keywords:** associations, hierarchical plan, rule learning, multiple cognitive processes, multiple-item memory, stimulus-response learning, timing, internal clock

## Introduction

“*Causality is a constraint common to all ecological niches.”*

*Macphail*, *1987*

Macphail (1987) claimed that all vertebrate nervous systems rely on detecting, encoding, and acting upon causality, and that there are no differences in intelligence between vertebrate species. The latter claim constitutes what is widely described as the “null hypothesis.” We examine the null hypothesis from the perspective of how vertebrates learn based on the order of events—that is, we will examine the ubiquity and foundations of sequential learning and memory in vertebrate behavior. The claim will be that several neurobehavioral systems subserve vertebrate sequence learning and that these and perhaps other systems together simplify encoding environmental complexity during learning and provide the foundation for performing complex but highly organized behavior.

What constitutes “causality” as coded by vertebrate brains? From an animal's perspective, behavior is inherently sequential and relevant events in the environment occur in probabilistic relationships with behavior. In the laboratory, these relationships may be highly constrained, as in Pavlovian conditioning, instrumental conditioning, and even more complex cognitive or neuroscience-oriented paradigms employing animals as complex as humans. This fact does not imply that the foundations of vertebrate behavior depend on a single underlying mechanism, though it should be no surprise that associative theory is a powerful approach to understanding and describing such behavior. On the contrary, much evidence suggests that vertebrate behavior is the result of multiple complementary systems that converge, interact, and often compete. These systems produce the remarkably adaptive and complex behavior befitting the remarkably diverse and complex environments in which vertebrates live. Yet, despite the diversity of scenarios in which behavior is played out, causality is universally available for organisms to exploit to survive and perpetuate the species. We propose that non-human vertebrates, like humans, abstract representations of simple causal relationships between events from complex environments, that is, they encode “simplicity from complexity.” Furthermore, vertebrates may share separate interacting systems for different types of sequential information.

Note that the critical and most challenging test of MacPhail's null hypothesis claim is *not* that the simplest processes are conserved “upward” to the most complex vertebrates, but that the most complex processes can be observed when we look “downward” toward the simplest vertebrates. To be explicit, we ask whether vertebrates in general extract “simplicity from complexity” through common learning mechanisms and neural substrates. As a start, this is the question we examine directly comparing humans, rats, mice, and pigeons. We conclude that additional evidence is needed to confirm our speculations regarding the generality of learning consisting of extracting “simplicity from complexity” in all vertebrates, but that Macphail (1987) was not far wrong in proposing that common processes may underlie vertebrate cognitive abilities though not necessarily resulting in equivalent capacities.

## What Cognitive Mechanisms are Common To Vertebrate Species?

Lashley (1951) rejected the notion that sequential behavior was accounted for by simple reflex chaining and argued instead for cognitive encoding of hierarchical plans. This notion contributed to the development of cognitive research in both human and non-human animals which continues to this day. Recently, Rosenbaum et al. (2007) shifted the theoretical concern from Lashley's focus on the nature of encoded sequence structures to identifying and describing the processes that contribute to the emergence of sequence structures in behavior. According to their view, even individual component movements are controlled by hierarchically organized plans (Rosenbaum et al., 2007).

Our work on non-human animal sequential rule abstraction, learning, and memory (retention) has also been motivated by Lashley's insights regarding the human capacity to use mental representations of pattern structures to plan ahead. As Reber (1993) and others have shown, plans need not be conscious or verbal. Early on, non-verbal pattern learning methods were developed to study sequence learning in rats (Hunter, 1920; Capaldi et al., 1966; Hulse, 1978), in monkeys (Straub et al., 1979; Straub and Terrace, 1981; Terrace, 1987), and in humans (Restle, 1970, 1973; Restle and Brown, 1970a,b). We developed a more general Serial Multiple Choice (SMC) task to analyze how rats, mice, pigeons, and humans learn complex sequential patterns of responses. A typical method allows animals to respond to circular arrays of 8 items, for example, a circular array of 8 manipulanda, one each on the walls of an octagonal chamber, or a circular array of 8 nosepoke locations on a touchscreen. In both cases, rats learn long but highly-organized patterns of responses on the manipulanda (e.g., Fountain et al., 2012; Garlick et al., 2017). Evidence from a variety of studies using this task indicate that all these species employ multiple concurrent cognitive processes to encode and produce complex sequential behavior (Fountain, 2008; Fountain et al., 2012). In the SMC task, the animal learns to make responses in a circular array (8-walled chamber or a circular touchscreen array). The required sequence of responses is typically a highly organized serial pattern of responses that may be characterized by multi-level hierarchical organization and “exceptions-to-the-rule.” Such patterns recruit multiple concurrent cognitive processes, namely, processes for encoding stimulus-response associations, timing/counting of events, and pattern structure including simple and higher-order rules (e.g., Muller and Fountain, 2010, 2016).

Furthermore, the learning and memory systems involved depend on different behavioral and neural systems as shown by dissociations observed in adulthood long after chronic adolescent drug exposure (Pickens et al., 2013; Renaud et al., 2015; Rowan et al., 2015; Renaud and Fountain, 2016; Sharp et al., 2019). Similar dissociations of cognitive behavioral systems can be seen in normal rats administered acute muscarinic cholinergic blockade (Pickens et al., 2013; Renaud et al., 2015; Rowan et al., 2015; Renaud and Fountain, 2016; Sharp et al., 2019). The SMC method in mice and rats produces data comparable to data in humans in an analogous task (Fountain and Rowan, 1995) and rats use multiple cognitive processes concurrently: rule-learning, stimulus-response (S-R) learning, discrimination learning, and multiple-item memory (Muller and Fountain, 2010, 2016). Fountain and Benson (2006) demonstrated chunking, rule-learning, and multiple-item memory when rats learned to anticipate the elements of two interleaved serial patterns. Mice show more limited abilities, but do show evidence of multiple concurrent learning processes (Fountain et al., 1999). Finally, pigeons in a comparable touchscreen task were able to abstract sequence structure (Garlick et al., 2017). Rats and, to a lesser degree, mice concurrently encode stimulus-response associations, time and count events, and abstract rules describing pattern structure (Muller and Fountain, 2010, 2016).

Taken together, these results strongly support the view that pigeons, mice, rats, and humans likely share multiple dissociable serial pattern learning and memory systems that encode multiple types of sequential information (Fountain et al., 2012). With reference to the underlying processes we have discussed in humans, rats, mice, and pigeons—namely, processes for encoding stimulus-response associations, timing/counting events, and rule-learning—are these common to all vertebrates? A broader survey of more species and new species-specific methods would be required to answer this question.

## Can A Single Associative Process Account For All Non-Human “Intelligence?”

In our attempts to characterize how rats learn to anticipate items in a sequence, in one approach we sought to use mathematical models to determine whether a single mechanism might account for all sequence learning in rats. One early success in this line of research that bears on this question was a mathematical model we used to determine whether a simple mathematical model based on simple associative principles could account for rat serial pattern learning for sequences of food quantities presented in a runway (Wallace and Fountain, 2002, 2003). We used a modified version of Metcalfe's Composite Holographic Associative Recall Model (CHARM; Metcalfe, 1990). In CHARM, items to be remembered are represented by vectors of random numbers, where each vector represents an item to be remembered. Our model based on CHARM is named the Sequential Pairwise Associative Memory (SPAM) model. SPAM used the same system of creating vectors of random numbers to represent food quantities, but vectors for different quantities varied in similarity to represent a range of food quantities from small to large. When the appropriate vectors for food quantities were stored for different sequential patterns, SPAM was able to simulate a full range of effects previously reported in the rat sequential learning literature of the time (Wallace and Fountain, 2002, 2003). On the other hand, that model and variations of it have so far failed to account for the variety of differences in learning phenomena revealed in how rats learn highly-structured sequences (Muller and Fountain, 2010, 2016). Nevertheless, the failure of one model, no matter how successful within a single domain yet failing in another, does not preclude the possibility that it might be possible to develop a successful single-process model that would be consistent with Macphail's claim.

Some aspects of cross-species behavioral comparisons of rats, mice, and pigeons (Fountain et al., 1999; Kelley and Rowan, 2004; Garlick et al., 2017) do not easily fit within the SPAM framework. SPAM does not account for several very robust aspects of serial pattern learning in rats in the SMC task. For example, a large body of our work indicates that independent processes mediate different types of learning via dissociable systems that operate concurrently for encoding simple associations, serial position, and lower- and higher-order rule structure (Muller and Fountain, 2010, 2016). These observations suggest that much research is needed to determine whether the same patterns of results would be observed in species other than the species we have already examined, namely, humans, rats, mice, and pigeons.

## The Role and Power of “Simple Associations,” “Hierarchical Plans,” and “Implicit Knowledge” in Vertebrate Cognitive Capacities

One conclusion we draw from the foregoing is that Lashley (1951) was correct to reject simple associative chaining accounts of sequential behavior. Lashley argued instead for a more cognitive account proposing that humans encoded and used hierarchical plans based on sequence structure and grouping which now seem fundamental to an analysis of animal sequence learning (Fountain et al., 2000; Muller and Fountain, 2010, 2016). Hierarchical plans, whether implicit or explicit, may be more fundamental than one might suppose given that even 8- to 10-month-old infants “exhibited sensitivity to the difference between hierarchical and non-hierarchical structure” and that the ability “to perceive, learn, and generalize recursive, hierarchical, pattern rules emerges in infancy” (Lewkowicz et al., 2018). The foregoing suggests that rule-learning in infancy must be implicit, and perhaps non-human vertebrates in general likewise can learn highly-organized implicit structures like those we have observed in pigeons, mice, and rats. This notion of hierarchical organization in behavior also unites our conceptions of behaviors as diverse as foraging (Feeney et al., 2011), bird song production and perception (e.g., Cazala et al., 2019), and sequential behavior (Swartz et al., 1991, 2000; Terrace and Chen, 1991a,b; Swartz and Himmanen, 2002; Suge and Okanoya, 2010; Spierings et al., 2015; Ramkumar et al., 2016). We go further to claim that vertebrate behavioral systems in their diversity encode different responses or different types of information, including complex associations, number (Brannon and Terrace, 2002), and time via internal clock processes (Tucci et al., 2014).

Macphail (1987) argued that all vertebrate nervous systems rely on detecting, encoding, and acting upon causality, and that there are no differences in intelligence between vertebrate species. We have described how a range of studies across paradigms and a variety of species support the view that complex learning processes may very well be broadly or even universally available to vertebrates. A challenge for the field is to develop experimental paradigms for assessing potentially common mechanisms in diverse species. At a foundational level, Macphail's claim continues to challenge all empiricists and theorists to consider the power of even simple neural systems to account for animals' ability to encode simplicity in terms of neural representation from the complexity of the surrounding environmental milieu.

## Author Contributions

All authors contributed significantly to background reading, organization of the paper, and writing.

## Conflict of Interest

The authors declare that the research was conducted in the absence of any commercial or financial relationships that could be construed as a potential conflict of interest.
